# Dopaminergic Modulation of Prospective Memory in Parkinson’s Disease

**DOI:** 10.1155/2008/310437

**Published:** 2008-04-11

**Authors:** Alberto Costa, Antonella Peppe, Livia Brusa, Carlo Caltagirone, Ilaria Gatto, Giovanni Augusto Carlesimo

**Affiliations:** ^1^I.R.C.C.S. Fondazione Santa LuciaRomaItaly; ^2^Ospedale S. EugenioRomaItaly; ^3^Clinica NeurologicaUniversità di Roma "Tor Vergata"RomeItaly

**Keywords:** Prospective memory, Parkinson’s disease, dopamine

## Abstract

Growing interest is present in literature on the study of prospective memory functioning in Parkinson’s disease (PD). Current data indicate that prospective memory may be impaired in PD and a relationship with general executive dysfunctioning has been suggested. However, although the dopamine dependency of cognitive dysfunction in PD has been widely investigated, poor is known on the dopaminergic modulation of PM. In the present study we explored the effect of acute administration of levodopa on the performance of a PD sample (*n* = 20) in a time-based prospective memory task. PD patients were evaluated in the morning after a 12-hour therapy wash-out in two experimental conditions: i) after levodopa assumption (“on”); (ii) without drug administration (“off”). The experimental task required to execute three uncorrelated actions after 10' for three consecutive trials. Distinct scores for the spontaneous recall of the intention to perform the actions (prospective component) and for the correct execution of the actions (retrospective component) have been computed. Results showed that in the “off”condition PD patients were selectively impaired on the prospective component of the task. However, L-dopa administration significantly improved PD patients’ performance actually restoring the prospective memory deficit. These results support a critical role of dopaminergic modulation in prospective memory processes in PD patients, possibly through the replacement of dopamine levels in fronto-striatal pathways.

## 1. Introduction

Prospective memory (PM)is defined as the ability to effectively comply with a planned action at a certain time (i.e., *time-based PM*) or when some external event occurs (i.e., *event-based PM*). It is generally held [1] that PM is composed of two distinct components: one that allows remembering the intention to perform some action at the appropriate moment (prospective component), the other that allows recalling the specific actions to be performed (retrospective component). Behavioural data collected in normally aged individuals and in patients with focal brain damage suggested that attentional and executive processes are particularly involved in supporting the cognitive operations that characterise the prospective component of the task; instead, the retrospective component would be mainly mediated by the same declarative memory system that is also responsible for the retrieval of previous facts or events [2,3]. From a neurobiological perspective, it seems that frontal lobes are particularly implicated in the implementation of PM processes. As a matter of fact, patients with focal lesions of the frontal lobes are less efficient in the ability to perform PM tasks [4]. Furthermore, recent PET and fRMI studies in healthy subjects showed that anterior and dorsolateral regions of prefrontal cortex play a crucial role in the genesis and retention of prospective intentions [5,6].

The presence of cognitive deficits involving the domain of executive functions is a frequent report in pa-

ISSN 0953-4180/08/$17.00 © 2008 – IOS Press and the authors. All rights reserved tients suffering from Parkinson's disease (PD). Some executive abilities, such as updating, set-shifting and set-maintaining and planning, have been shown to be early impaired in the disease course [7,8]. Some studies also documented the presence of memory disorders in these patients [9], that seems to be mainly owed to a specific difficulty in self-initiating remembering [10]. Coherently with these evidences, recent data documented that PM may be impaired in PD patients too [11–13]. In particular, in a recent study [13] we investigated the ability of a group of 23 non-demented PD patients as compared to a group of 25 healthy subjects to execute some planned actions at a certain time (time-based task) or after the ring of a timer (event-based task). We found that PD patients were impaired in the prospective component of the time-based but not of the event-based task. Differently from previous studies, our sample of PD patients was also impaired in remembering the specific actions to be performed. Finally, a trend toward a significant association was found between poor performances on some measure of executive and short-term memory functioning and the PM impairment.

Several studies investigated the dopamine dependency of cognitive dysfunctioning in PD [14]. Although the results from these studies are not univocal, they suggest that cognitive functions mainly mediated by a neural circuitry entailing the dorsolateral prefrontal cortex and the caudate nucleus could benefit from early dopamine medication [7,8]. However, to date, the relationship between dopamine therapy and PM functioning has not been investigated in PD. The present study was aimed at assessing the possible role of dopamine depletion in the genesis of the PM impairment in patients with PD.

## 2. Subjects and methods

### *2.1. Subjects*

Twenty individuals affected by idiopathic PD and 15 healthy subjects (HS) participated in the study. The diagnosis of idiopathic PD was made by an expert neurologist based on $i)$ the presence of at least two of the four cardinal symptoms; *ii*) good chronic response to L-dopa treatment [15]. Exclusion criteria for the PD group included $i)$ Hohen and Yahr's [16] score $>2.5$; *ii*) presence of dementia; *iii*) presence of severe systemic or metabolic diseases; *iv*) marked cortical and subcortical atrophy and/or ischemic vascular lesions on CT and/or MRI scans; $v)$ a Major Depressive Disorder; *vi*) presence in the history of other neurological disorders, head trauma and substance abuse; *vii*) severe functional impairment of autonomic nervous system; *viii*) use of other active central nervous system therapies. Clinical and demographic characteristics of the PD and HS groups are reported in Table 1.

### *2.2. Methods*

The PD subjects were evaluated in the morning following therapy withdrawal of at least 12 hours. In a first condition, the PM task was administered to PD patients 20–30 min after oral administration of 200 mg of L-dopa ("on" therapy condition); in the other condition the PM task was performed without any prior drug administration ("off" therapy condition). The two treatment conditions were administered about two weeks apart and the order was randomised across subjects. The HC group performed the task only once, without any drug administration.

The overall experimental PM procedure consisted of three consecutive trials. At the beginning of each trial, the examiner required the subject to execute three functionally unrelated actions (e.g., telling the examiner to turn on the computer, the patient writing his own name on a sheet of paper, replacing the telephone receiver) following a 10 min. interval. During the interval the subject was engaged in tasks evaluating attention, executive functions and short-term memory (i.e., the Stroop test [18], Attentive Matrices [19], the Trail Making test [20] and the Digit span [21]). If the subject autonomously started to perform some action at the established time the examiner recorded the actions actually carried out, regardless of whether or not they followed the order indicated by the examiner. If the subject did not spontaneously engage in any action the examiner reminded him: "Do you remember that at this point you were supposed to do something?" In the case of an affirmative response, the examiner recorded the number of actions carried out correctly. Two separate scores were then computed, i.e., for the recall of the intention to perform the actions (prospective component) and for the correct execution of the actions (retrospective component). Note that since if the subjects did not begin to perform any of the actions then the examiner solicited them to remember the specific actions to be performed, the scores attributed for the prospective and retrospective components of the task were reciprocally independent, i.e., a failure to prospectively remember the intention did not influence the score obtained on the task of retrospectively remembering the specific actions to be performed.

Table 1
Demographic and clinical characteristics of individuals in the PD and HS groups. Results of ANOVAs are also reported

| Demographic and clinical variables | PD subjects $N = 20$ | HS $N = 15$ | F (1,33) |
|---|---|---|---|
| M/F | 13/7 | 9/6 | |
| | Mean $\pm$ SD | Mean $\pm$ SD | |
| Age (yrs) | 60.3 $\pm$ 9.5; | 61.1 $\pm$ 7.0; | 0.08 |
| | Range: 43–74 | Range: 49–70 | |
| Formal education (yrs) | 10.0 $\pm$ 3.2 | 9.7 $\pm$ 3.6 | 0.07 |
| Age at disease onset (yrs) | 54.2 $\pm$ 11.4 | | |
| Unified Parkinson's Disease Rating Scale [17] ("off" therapy condition) | 24.5 $\pm$ 9.2 | | |
| Disease duration (yrs) | 6.1 $\pm$ 6.1 | | |

Table 2
Performance score achieved by PD patients and HS on the prospective (i.e., the number of retrieved intentions) and retrospective (i.e., the number of correctly recalled actions) components of the experimental task. As for PD patients, scores achieved after the dopamine therapy withdrawal ("off" therapy condition) and after the acute administration of levodopa ("on" therapy condition) are presented

| | PD patients "off" therapy condition | PD patients "on" therapy condition | HS | PD patients "off" therapy condition vs. HS Man Whitney U test | PD patients "off" vs. "on" therapy conditions Friedman ANOVA |
|---|---|---|---|---|---|
| | | Mean $\pm$ SD | | | |
| Prospective component | 1.7 $\pm$ 1.1 | 2.4 $\pm$ 0.8 | 2.7 $\pm$ 0.4 | $z = -2.97$; $p = 0.005$ | $\chi 2$ (df $= 1$) =6.40; $p = 0.011$ |
| Retrospective component | 8.1 $\pm$ 1.1 | 8.3 $\pm$ 1.0 | 7.7 $\pm$ 1.2 | $z = 0.93$; $p =$ n.s. | $\chi 2$ (df =1) $=$ 0.00; $p = n.s.$ |

## 3. Results

The main results of the study are summarized in Table 2. As shown in the Table, PD patients in "off" therapy condition obtained significantly lower scores than HS in the prospective but not in the retrospective component of the PM task. The administration of L-dopa significantly improved PD participants' accuracy in the retrieval of the intention in the experimental task while no significant difference was found as a function of pharmacological treatment for the ability to remember the specific actions to be performed.

The score obtained on the Unified Parkinson's Disease Rating Scale [17] significantly decreased passing from the "off" to the "on" therapy condition (24.5 $\pm$ 9.2, and 12.1 $\pm$ 4.5 respectively; t (df $=$ 19) $=$ 9.23; $p < 0.001$), thus confirming that L-dopa treatment was effective in ameliorating motor symptoms of PD patients.

As for the intercurrent tasks, in "off" therapy condition PD patients performed significantly worse than HCs on the Digit Span forward and on the word and color-word section of the Stroop test ($p < 0.05$). However, no significant ameliorative effect of L-dopa on the PD patients' performance on these tasks was found. Spearman's Rho correlations analyses, executed in the PD group to evaluate the relationship between the rate of improvement in the prospective component of the PM task and the rate of improvement in the intercurrent tasks after L-dopa administration, showed a significant association between the rate of improvement observed in the prospective memory task and the reduction in the number of errors made in the color-word section of the Stroop test ($p = 0.017$). No other significant effect was found.

## 4. Discussion

The present study was aimed at evaluating the relationship between dopamine treatment and PM functioning in a group of PD patients. The main result of the study is the ameliorative effect produced by L-dopa administration on PD patients' performance in the prospective component of a time-based PM task. In fact, the impaired retrieval of the intention to perform planned actions which was evident in the PD patients following withdrawal of the L-dopa therapy, significantly improved after the acute administration of L-dopa. Moreover, the rate of improvement observed in the PM task after L-dopa intake was associated to the rate of improvement disclosed by the same patients in the ability to inhibit the interference exerted by automatic processes as in the Stroop test.

Comprehensively, our findings are in line with previous studies suggesting a critical role for dopaminergic alterations in the genesis of cognitive deficits in PD [8,14]. In particular, since, as previously stated, there is evidence that dorso-lateral and rostral areas of the prefrontal cortex are particularly involved in mediating PM processes [4–6], our data seem to indicate that a replacement of dopamine levels in fronto-striatal pathways was the basic mechanism of improved performance in the prospective memory task in our PD patients.

In conclusion, this is the first study aimed at investigating the dopaminergic modulation of PM in PD. Further studies using, for instance, functional neuroimaging techniques to directly explore the neural changes as a function of levodopa treatment, could add some valuable information on this issue.
